# Messung des krankheitsbezogenen Wissens von Patient*innen mit axialer Spondyloarthritis – Entwicklung und Anwendung des G-ASKQ7-Fragebogens

**DOI:** 10.1007/s00393-024-01584-x

**Published:** 2024-11-13

**Authors:** Louis Schuster, Jörg Henes, Matthias Diener, Felix Mühlensiepen, Ioana Andreica, Axel Hueber, Georg Schett, Johannes Knitza

**Affiliations:** 1 Friedrich-Alexander-Universität Erlangen-Nürnberg (FAU) Erlangen-Nürnberg, Erlangen-Nürnberg, Deutschland; 2 Medizinische Klinik 3 – Rheumatologie und Immunologie, Universitätsklinikum Erlangen, Ulmenweg 18, 91054 Erlangen, Deutschland; 3 Zentrum für interdisziplinäre Rheumatologie, Immunologie und Autoimmunerkrankungen (INDIRA) und Innere Medizin II, Universitätsklinik Tübingen, Tübingen, Deutschland; 4 Digital Rheuma Lab, Berlin, Deutschland; 5 Zentrum für Versorgungsforschung, Fakultät für Gesundheitswissenschaften, Medizinische Hochschule Brandenburg, Rüdersdorf bei Berlin, Deutschland; 6 Ruhr-Universität Bochum, Bochum, Nordrhein-Westfalen Deutschland; 7 Rheumazentrum Ruhrgebiet, Herne, Nordrhein-Westfalen Deutschland; 8 Abteilung Rheumatologie, Klinik für Innere Medizin 5, Klinikum Nürnberg, Universitätsklinik der Paracelsus Medizinischen Universität, Nürnberg, Deutschland; 9 Institut für Digitale Medizin, Philipps Universität Marburg und Universitätsklinikum Gießen und Marburg, Baldingerstr., 35043 Marburg, Deutschland

**Keywords:** Gesundheitskompetenz, Patientenschulung, Axiale Spondylarthritis, Versorgungsforschung, Selbstmanagement, Health competence, Patient education, Axial spondyloarthritis, Health services research, Self-management

## Abstract

**Hintergrund/Ziel:**

Patientenedukation ist integraler Bestandteil der Behandlung von Patient*innen mit axialer Spondyloarthritis (axSpA). Allerdings sind die verfügbaren validen Messinstrumente zur Erhebung des krankheitsbezogenen Wissens veraltet und im Falle der axSpA nur in englischer Sprache verfügbar. Die Ziele dieser Studie waren daher (1) die Entwicklung eines geeigneten deutschsprachigen Instruments zur Messung krankheitsspezifischen Wissens bei axSpA-Patient*innen und (2) die Pilotierung dieses Instruments.

**Methoden:**

Verfügbare Messinstrumente speziell für die axSpA wurden von einem Expertengremium geprüft, und ein Entwurf des aktuellen G‑ASKQ7 wurde abgestimmt. Nach Evaluation der Augenscheinvalidität und entsprechenden Anpassungen wurden erwachsene konsekutive axSpA-Patient*innen der rheumatologischen Hochschulambulanz des Universitätsklinikums Erlangen gebeten, den Fragebogen auszufüllen. Es wurden Korrelationen zwischen dem erreichten Summenscore und weiteren erhobenen Parametern (Alter, Geschlecht und Krankheitsdauer) untersucht.

**Ergebnisse:**

Der entwickelte G‑ASKQ7-Fragebogen umfasst 7 Fragen, wurde als leicht verständlich bewertet und innerhalb von durchschnittlich 6,3 ±2,0 min ausgefüllt; 65 axSpA-Patient*innen (36 Frauen; mittleres Alter: 45,3 ± 12,4 Jahre; mittlere Erkrankungsdauer: 8,6 ±7,3 Jahre) beantworteten den finalen Fragebogen. Der G‑AKSQ7-Mittelwert lag bei 17,8/25 und war bei Frauen (19,0/25) signifikant höher als bei Männern (16,3/25) (*p* = < 0,05). Am häufigsten wurde Frage 7 (79,3 %) mit Hinblick auf das Selbstmanagement der Erkrankung korrekt beantwortet. Frage 4 mit Blick auf die Messung der Krankheitsaktivität wurde am schlechtesten beantwortet (55,8 %). Es konnte keine signifikante Korrelation zwischen der Krankheitsdauer (*p* = 0,57; r = 0,07; Pearson-Korrelationstest) oder dem Alter (*p* = 0,67; r = −0,05; Pearson-Korrelationstest) und dem krankheitsbezogenen Wissen festgestellt werden.

**Diskussion:**

Der G‑ASKQ7 bietet erstmals ein deutschsprachiges Instrument zur Ermittlung des krankheitsspezifischen Wissens von axSpA-Patient*innen. Durch eine routinemäßige Erhebung mittels G‑ASKQ7 kann der individuelle Wissensbedarf von Patient*innen niedrigschwellig ermittelt werden, und Edukationsinhalte können gezielt auf Patient*innen abgestimmt werden. Eine kontinuierliche Aktualisierung und weitere Evaluationsstudien sind erforderlich.

**Zusatzmaterial online:**

Die Online-Version dieses Beitrags (10.1007/s00393-024-01584-x) enthält den G‑ASKQ7-Fragebogen.

## Abstract (en)

**Background and objective:**

Patient education is an integral component of the treatment of axial spondyloarthritis (axSpA). However, the validated tools currently available for assessing disease-related knowledge are outdated and, in the case of axSpA, only available in English. The aims of this work were therefore (1) to develop a German-language tool for assessing disease-specific knowledge among axSpA patients and (2) to assess this tool in a pilot study.

**Methods:**

Available axSpA-specific tools were assessed by a panel of experts and a draft of the current G‑ASKQ7 questionnaire was agreed upon. After evaluation of face validity and subsequent adaptation, consecutive adult axSpA patients of a university hospital (Universitätsklinikum Erlangen) outpatient rheumatology department were asked to complete the questionnaire. Correlation between the patients′ overall scores and other collected parameters (age, sex, and disease duration) were investigated.

**Results:**

Patients rated the developed G‑ASKQ7 questionnaire comprising seven questions as easy to understand and completed it within an average of 6.3 ± 2.0 min. The final questionnaire was completed by 65 axSpA patients (36 women; mean age 45.3 ± 12.4 years; mean disease duration 8.6 ± 7.3 years). The average G‑AKSQ7 score was 17.8/25, with significantly higher scores among women (19.0/25) than men (16.3/25; *p* ≤ 0.05). The question most often answered correctly (79.3 %) was question 7 concerning disease self-management, whereas question 4 on measurement of disease activity received the most incorrect answers (55.8 %). No significant correlation between disease duration (*p* = 0.57; Pearson′s correlation coefficient, r = 0.07) or age (*p*  = 0.67; r = −0.05) and disease-specific knowledge was found.

**Conclusion:**

The G‑ASKQ7 represents the first German-language instrument for measuring disease-specific knowledge among axSpA patients. Routine application of this questionnaire would enable low-threshold assessment of an individual patient′s knowledge requirements and thus provision of appropriate educational information. Continuous updates and further evaluation studies are required.

Die axiale Spondyloarthritis (axSpA) zählt mit einer geschätzten Prävalenz von 0,32–0,5 % zu den häufigsten Erkrankungen im entzündlich rheumatischen Formenkreis in Deutschland [1]. Gemäß der aktuellen S3-Leitlinie „Axiale Spondyloarthritiden einschließlich ankylosierende Spondylitis und Frühformen“ sowie den Empfehlungen der European Alliance of Associations for Rheumatology (EULAR) wird die Bedeutung des Patientenwissens zur Verbesserung des Krankheitsmanagements bei gleichzeitiger Kostenreduktion betont [7]. Der Wissensstand unter den Betroffenen in Deutschland ist nicht bekannt und kann aufgrund veralteter Messinstrumente, die im Falle der axSpA nur auf Englisch vorliegen, derzeit nicht standardisiert ermittelt werden [9]. Ziel dieser Studie war daher (1) die Entwicklung eines geeigneten deutschsprachigen Messinstruments zur Erhebung des krankheitsspezifischen Wissens von axSpA-Patient*innen und (2) die Erprobung dieses Instruments in einer kleinen Kohorte von axSpA-Patient*innen.

## Methode

### Entwicklung des Fragebogens

Es erfolgte eine Recherche und Bewertung der existierenden Instrumente, und es wurde ein Panel, bestehend aus Patient*innen und Rheumatolog*innen gebildet, um die vorhandenen Instrumente kritisch zu diskutieren und einen deutschsprachigen Fragebogen zu konsentieren.

### Inhaltliche Validierung und Finalisierung: Prüfung der Augenscheinvalidität und Machbarkeit

Die inhaltliche Validierung wurde nach den Grundsätzen der kognitiven Befragung durchgeführt [2]. In unserer Studie wurde diese Technik z. B. angewandt, indem axSpA-Patient*innen gefragt wurden: „Was kommt Ihnen in den Sinn, wenn Sie dies lesen?“, „Was ist Ihnen bei der Beantwortung dieser Frage aufgefallen?“ oder „Fiel es Ihnen leicht oder schwer, diese Frage zu beantworten?“. Die Teilnehmer wurden also gebeten, den Fragebogen durchzugehen und dabei laut zu denken und die Fragen und Optionen zu kommentieren. Diese Erhebung wurde durch JK durchgeführt, der ebenfalls Zeitaufwand, Zögern und Ausdrucksweise dokumentierte. Es wurde eine gezielte Auswahlstrategie angewandt, um eine demografische Vielfalt zu erreichen, indem wir Teilnehmer beider Geschlechter mit unterschiedlichem Alter und unterschiedlicher Krankheitsdauer einschlossen. Anhand des Patient*innen-Feedbacks wurde der Fragebogen anschließend finalisiert.

### Anwendung des G-ASKQ7

Der finale Fragebogen (German Axial Spondyloarthritis Knowledge Questionnaire; G‑ASKQ7) wurde durch konsekutive erwachsene axSpA-Patient*innen der Rheumatologischen Hochschulambulanz des Universitätsklinikums Erlangen pilotiert, und es wurden Korrelationen zwischen dem erreichten Summenscore und anderen erhobenen Parametern (Alter, Geschlecht und Krankheitsdauer) untersucht.

## Ergebnisse

### G-ASKQ7 Fragebogenentwicklung

Als Grundlagen wurden 2 Publikationen identifiziert, der 1998 von Lubrano et al. veröffentlichte Fragebogen [9] und eine rezentere Studie von Beauvais et al., die essenzielle axSpA-Fragen ermittelte [4]. Die Erkenntnisse dieser Veröffentlichung wurden im Board diskutiert und anschließend von JK zu einem Entwurf ausgearbeitet. Die Übersetzung der vorhandenen englischen Fragen basierte auf den Methoden des International Quality of Life Assessment (IQOLA) und impliziert Vorwärts- und Rückwärtsübersetzungen [5]. Der G‑ASKQ7-Fragebogen (s. Zusatzmaterial online) deckt die durch Beauvais identifizierten Kernthemen ab [3] und umfasst insgesamt 7 Fragen, 48 Antwortoptionen mit 25 enthaltenen korrekten Antworten. Zur Berechnung des Beantwortungserfolgs wird ein einfacher Summenscore der korrekten Antworten (s. Zusatzmaterial online) gebildet.

### Inhaltliche Validierung und Finalisierung

Zehn axSpA-Patient*innen (5 weiblich, 5 männlich; mittleres Alter 47,2 ± 18,2 Jahre) pilotierten den G‑ASKQ7-Entwurf. Der Fragebogen wurde als leicht verständlich aufgefasst, und es wurden letztlich nur 2 Rechtschreibfehler korrigiert. Die durchschnittliche Bearbeitungszeit lag bei 6,3 ± 2,0 min.

### G-ASKQ7-Testergebnisse

Insgesamt haben 65 konsekutive axSpA-Patient*innen den finalen Fragebogen zwischen Juni und Dezember 2022 beantwortet, davon waren 36 Frauen (55 %). Das Durchschnittsalter der Befragten betrug 45,3 ± 12,4 Jahre, und die durchschnittliche Erkrankungsdauer lag bei 8,6 ± 7,3 Jahren; 80 % der Proband*innen beantworteten mehr als 50 % der gestellten Fragen richtig. In Tab. 1 sind alle Behauptungen mit ihrer Ankreuzhäufigkeit aufgeführt. Die durchschnittliche Antwortgenauigkeit über alle Fragen hinweg betrug 17,8 von 25 möglichen richtigen Zuordnungen. Wir beobachteten deutliche themenspezifische Unterschiede des Wissens: Frage 4 zu den Verfahren zur Beurteilung einer aktiven Erkrankung wurde von den Teilnehmenden im Durchschnitt am schlechtesten beantwortet (55,8 %, Mittelwert: 2,23). Frage 7, die auf den Umgang mit der Erkrankung abzielt, wurde hingegen am besten beantwortet (79,3 %, Mittelwert: 2,38).

**Tab. 1:** Häufigkeit des Ankreuzens der jeweiligen Aussagemöglichkeiten

Behauptung	Wahrheitsgehalt	Zutreffend markiert (%)
**Frage 1: Axiale Spondylarthritis:**		
a) Ist eine Infektionskrankheit	Falsch	9
b) Die Ursache ist nicht bekannt	Korrekt	54
c) Kann vererbt werden	Korrekt	69
d) Tritt am häufigsten im hohen Alter auf	Falsch	18
e) Wird durch sportliche Betätigung oder Verletzung verursacht	Falsch	2
f) Es handelt sich um eine chronische Krankheit	Korrekt	86
g) Ich weiß es nicht	Unzureichend	18
**Frage 2: Axiale Spondylarthritis:**		
a) Verursacht Gelenkentzündungen	Korrekt	88
b) Die ersten Beschwerden sind nicht unbedingt im Rücken	Korrekt	74
c) Verschlimmert sich immer bei kaltem Wetter	Falsch	34
d) Tritt wahrscheinlicher bei Patienten auf, die HLA-D4-positiv sind	Falsch	3
e) Ist eine heilbare Krankheit	Falsch	3
f) Tritt wahrscheinlicher bei Patienten auf, die HLA-B27-positiv sind	Korrekt	62
g) Ich weiß es nicht	Unzureichend	15
**Frage 3: Axiale Spondylarthritis:**		
a) Kann das Auge und die Achillessehne betreffen	Korrekt	68
b) Kann ohne angemessene Behandlung zu einer Verschmelzung von Knochen in der Wirbelsäule führen	Korrekt	75
c) Kann Schmerzen und Steifheit im Rücken verursachen	Korrekt	86
d) Erhöht das Risiko einer Krebserkrankung	Falsch	11
e) Der Krankheitsverlauf umfasst Phasen mit Schüben und Remission	Korrekt	83
f) Röntgenbilder können bei Diagnose unauffällig sein	Korrekt	65
g) Ich weiß es nicht	Unzureichend	15
**Frage 4: Bitte wählen Sie die vier Verfahren aus, die eingesetzt werden, um zu beurteilen, wie aktiv die axiale Spondylarthritis ist:**		
a) Cholesterinspiegel	Falsch	8
b) ESR (Erythrozytensedimentationsrate)	Korrekt	37
c) Blutbild	Falsch	65
d) CRP (C-reaktives Protein)	Korrekt	55
e) MRT (Magnetresonanztomographie)	Korrekt	80
f) Validierte Patientenfragebögen	Korrekt	51
g) Ich weiß es nicht	Unzureichend	22
**Frage 5: Axiale Spondylarthritis:**		
a) Es gibt nur ein Medikament für diese Krankheit	Falsch	8
b) Die medikamentöse Therapie ist der einzige Ansatz zur Kontrolle der Krankheit	Falsch	43
c) Es kann einige Wochen dauern, bis die medikamentöse Therapie Wirkung zeigt	Korrekt	91
d) Verschiedene NSAIDs können helfen und sollten ausprobiert werden, um Schmerzen und Entzündungen zu reduzieren	Korrekt	80
e) Zur Überwachung der Arzneimittelsicherheit sind regelmäßige Blutuntersuchungen erforderlich	Korrekt	83
f) NSAIDs (z. B. Ibuprofen) können gastrointestinale Nebenwirkungen wie Gastritis hervorrufen. Schwarzer Stuhl kann ein Symptom für Magen-Darm-Blutungen sein	Korrekt	69
g) Falls NSAIDs nicht wirksam sind, können Biologika eingesetzt werden	Korrekt	63
h) Biologika können bei schweren, behandlungsbedürftigen Infektionen (z. B. Lungenentzündung, Zahnabszesse usw.) eingesetzt werden	Falsch	3
i) NSAIDs bergen kein Risiko für Herz-Kreislauf-Erkrankungen, z. B. Bluthochdruck, und für Nierenfunktionsstörungen	Falsch	9
j) Ich weiß es nicht	Unzureichend	11
**Frage 6: Bitte wählen Sie die beiden korrekten Antworten zum Thema Impfung aus:**		
a) Lebendimpfstoffe sind unter Biologika normalerweise kontraindiziert (verboten)	Korrekt	58
b) Können Infektionen verhindern	Korrekt	63
c) Verschlimmern sehr oft Krankheitssymptome	Falsch	11
d) Ich weiß es nicht	Unzureichend	31
**Frage 7: Bitte wählen Sie die drei korrekten Antworten aus der folgenden Liste aus:**		
a) Bettruhe für den größten Teil des Tages ist die beste Lösung, wenn Ihr Rücken schmerzt und steif ist	Falsch	3
b) Übungen zu Hause, Krankengymnastik, Massagen und Dehnübungen sind hilfreich, um Wirbelsäulensteifigkeit entgegenzuwirken	Korrekt	97
c) Körperliche Aktivität und Training verschlimmern Müdigkeit (Fatigue)	Falsch	18
d) Müdigkeit (Fatigue) hat viele Ursachen: Entzündungen und Schübe, Medikamente, Lebensstil, Stimmung, Schlafstörungen	Korrekt	89
e) Es gibt eine individuelle Anfälligkeit für NSAIDs (z. B. Ibuprofen), und es sollte die niedrigste wirksame Dosis angestrebt werden	Korrekt	52
f) Ich weiß es nicht	Unzureichend	8

Die Korrelationen zwischen der erreichten Gesamtpunktzahl und anderen erhobenen Parametern waren ebenfalls Gegenstand der Untersuchung. Unsere Hypothese war, dass ein Zusammenhang zwischen der Erkrankungsdauer und dem Wissen über axSpA besteht. Allerdings konnte lediglich eine äußerst geringe Korrelation (*p* = 0,57; r = 0,07; Pearson-Korrelationstest) zwischen der Krankheitsdauer und dem Summenscore festgestellt werden. Eine Abhängigkeitsbeziehung ist daher unwahrscheinlich.

**Abb. 1:**
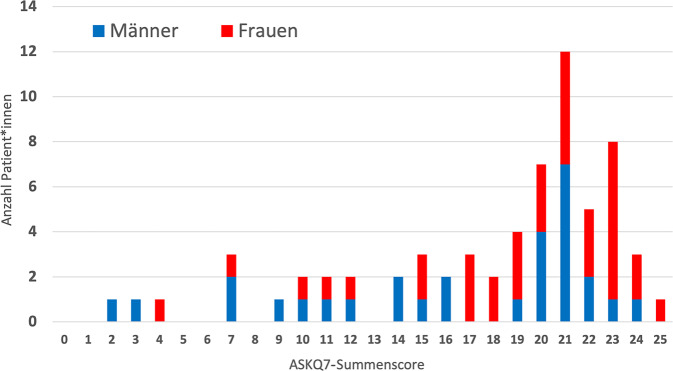
Verteilung des erreichten Summenscores entsprechend Geschlecht

Wir konnten auch keinen Zusammenhang zwischen dem Alter und dem erreichten Summenscore beobachten (*p* = 0,67; r = −0,05; Pearson-Korrelationstest). Frauen erreichten einen signifikant höheren Score (19,0/25) im Vergleich zu Männern (16,3/25), (*p* = < 0,05) mit einem schwach positiven Effekt (Cliff’s Delta = 0,27). Der erreichte Summenscore, aufgeteilt nach Geschlecht, ist in Abb. 1 dargestellt.

## Diskussion

Ziel der Arbeit war erstmals ein deutschsprachiges Messinstrument für das krankheitsspezifische Wissen von axSpA-Patient*innen zu entwickeln und zu erproben. Der G‑ASKQ7 ermöglicht es, verständlich, schnell und standardisiert das krankheitsspezifische axSpA-Wissen von Patient*innen zu ermitteln.

Die ersten Ergebnisse zeigen, dass der Kenntnisstand der Patientinnen und Patienten über die Krankheit axSpA gut, aber auch verbesserungswürdig ist. Der G‑ASKQ7 wurde im Durchschnitt etwas schlechter beantwortet als der englischsprachige Vergleich von 1998 (Mittelwert: Referenz [19,4]/G-ASKQ7 [17,8]). Im Gegensatz zum Referenzfragebogen wurde jedoch ein statistischer Zusammenhang zwischen Geschlecht und Wissensstand festgestellt [9]. Eine Verallgemeinerung dieser Aussage lässt das Ergebnis dieser Studie nicht zu.

Der G‑ASKQ7 könnte initial nach der Erstdiagnose oder bei späteren jährlichen „Check-up-Terminen“ genutzt werden, um standardisiert den Erfolg der empfohlenen Wissensvermittlung zu überprüfen [6]. Darüber hinaus ließe sich auf dieser Basis die weitere Wissensvermittlung – auch im Hinblick auf digitale Interventionen – gezielt planen [8].

Eine weitergehende Validierung des Fragebogens hinsichtlich Reliabilität, Anforderungsniveau oder Lesbarkeit wurde nicht durchgeführt, da bei der Erstellung des Fragebogens bereits konsentierte Fragen bzw. ein validierter Fragebogen als Vorlage verwendet wurden. Darüber hinaus wurde das allgemeine Bildungsniveau der Teilnehmenden nicht ermittelt, was sich ebenfalls auf die erreichte Punktzahl ausgewirkt haben könnte. Der Fragebogen sollte auch hinsichtlich der Formulierung bzw. Verständlichkeit der Aussagen weiter überarbeitet werden, da z. B. 65 % der Befragten fälschlicherweise eine Blutbilduntersuchung als Methode zur Bestimmung der Krankheitsaktivität angekreuzt hatten, diese aber ggf. aufgrund der Fachterminologie falsch zugeordnet wurde. Insgesamt sollten entsprechende Fragebögen kontinuierlich angepasst werden, wofür auch qualitative Stakeholder-Interviews herangezogen werden sollten [8]. In der Zwischenzeit wurde beispielsweise ein weiterer englischsprachiger Fragebogen publiziert, der jedoch mit 42 (Langversion) und 32 (Kurzversion) Items wesentlich länger ist als der G‑ASKQ7 [3]. Eine weitere Limitation besteht im gewählten Summenscore. Ein Vorteil des Instruments liegt in einfacher Handhabung. Allerdings führt die Auswahl falscher Antworten nicht zu einer Verringerung der erreichten Gesamtpunktzahl. Darüber hinaus sollte eine Folgestudie mit einer gezielten Wissensintervention und einer klaren Berechnung der Fallzahl durchgeführt werden, um auch die Responsivität des Instruments zu untersuchen.

## Fazit für die Praxis

- Der G‑ASKQ7 ermöglicht es, schnell und standardisiert das krankheitsspezifische axSpA-Wissen von deutschsprachigen Patient*innen zu ermitteln.
- Das krankheitsspezifische Wissen der axSpA-Patienten scheint gut, aber ausbaufähig.
- Eine kontinuierliche Weiterentwicklung, Aktualisierung und weitere Evaluationsstudien des G‑ASKQ7 sind notwendig.

## Supplementary Information

G‑ASKQ7-Fragebogen
