# Supplementary material for: Measurement of disease-related knowledge of patients with axial spondyloarthritis—Development and application of the G-ASKQ7 questionnaire
Source: Z Rheumatol. 2024 Nov 13;84(5):404–9. [Article in German] doi: 10.1007/s00393-024-01584-x (PMC12134029; doi:10.1007/s00393-024-01584-x)
Supplement: Supplementary file 1 — G‑ASKQ7-Fragebogen [file 393_2024_1584_MOESM1_ESM.pdf]

G-ASKQ7-Fragebogen

Wissen über axiale Spondylarthritis

Dieser Fragebogen behandelt die axiale Spondylarthritis, und wir bitten Sie, die Fragen auf der Grundlage Ihres Wissens über axiale Spondylarthritis zu beantworten.
Bitte beantworten Sie die Fragen auf der Grundlage Ihres allgemeinen Wissens über axiale Spondylarthritis und nicht bezogen darauf, wie sich die Krankheit auf Sie auswirkt.

Bitte beantworten Sie jede Frage und kreuzen Sie die Optionen an, die Sie für korrekt halten. Sie können mehr als eine Antwortmöglichkeit ankreuzen.

---

1. Bitte wählen Sie **drei korrekte** Aussagen aus den folgenden Optionen aus:
   Axiale Spondylarthritis:

   a. Ist eine Infektionskrankheit
   b. Die Ursache ist nicht bekannt
   c. Kann vererbt werden
   d. Tritt am häufigsten im hohen Alter auf
   e. Wird durch sportliche Betätigung oder Verletzungen verursacht
   f. Es handelt sich um eine chronische Krankheit
   g. Ich weiß es nicht
2. Bitte wählen Sie **drei korrekte** Aussagen aus den folgenden Optionen aus:
   Axiale Spondylarthritis:

   a. Verursacht Gelenkentzündungen
   b. Die ersten Beschwerden sind nicht unbedingt im Rücken
   c. Verschlimmert sich immer bei kaltem Wetter
   d. Tritt wahrscheinlicher bei Patienten auf, die HLA-D4-positiv sind
   e. Ist eine heilbare Krankheit
   f. Tritt wahrscheinlicher bei Patienten auf, die HLA-B27-positiv sind
   g. Ich weiß es nicht
3. Bitte wählen Sie **fünf korrekte** Aussagen aus den folgenden Optionen aus:
   Axiale Spondylarthritis:

   a. Kann das Auge und die Achillessehne betreffen
   b. Kann ohne angemessene Behandlung zu einer Verschmelzung von Knochen in der Wirbelsäule führen
   c. Kann Schmerzen und Steifheit im Rücken verursachen
   d. Erhöht das Risiko einer Krebserkrankung
   e. Der Krankheitsverlauf umfasst Phasen mit Schüben und Remissionen
   f. Röntgenbilder können bei Diagnose unauffällig sein
   g. Ich weiß es nicht

4. <u>Bitte wählen Sie die **vier Verfahren** aus, die eingesetzt werden, um zu beurteilen, wie aktiv die axiale Spondylarthritis ist:</u>

    a. Cholesterinspiegel
    b. ESR (Erythrozytensedimentationsrate)
    c. Blutbild
    d. CRP (C-kreatives Protein)
    e. MRI (Magnetresonanztomographie)
    f. validierte Patientenfragebögen
    g. Ich weiß es nicht

5. <u>Bitte wählen Sie **fünf korrekte** Aussagen aus den folgenden Optionen aus:</u>
   Axiale Spondylarthritis:

    a. Es gibt nur ein Medikament für diese Krankheit
    b. Die medikamentöse Therapie ist der einzige Ansatz zur Kontrolle der Krankheit
    c. Es kann einige Wochen dauern, bis die medikamentöse Therapie Wirkung zeigt
    d. Verschiedene NSAIDs können helfen und sollten ausprobiert werden, um Schmerzen und Entzündungen zu reduzieren
    e. Zur Überwachung der Arzneimittelsicherheit sind regelmäßige Blutuntersuchungen erforderlich.
    f. NSAIDs (z. B. Ibuprofen) können gastrointestinale Nebenwirkungen wie Gastritis hervorrufen. Schwarzer Stuhl kann ein Symptom für Magen-Darm-Blutungen sein.
    g. Falls NSAIDs nicht wirksam sind, können Biologika eingesetzt werden
    h. Biologika können bei schweren, behandlungsbedürftigen Infektionen (z. B. Lungenentzündung, Zahnabszess usw.) eingesetzt werden.
    i. NSAIDs bergen kein Risiko für Herz-Kreislauf-Erkrankungen, z. B. Bluthochdruck, und für Nierenfunktionsstörungen
    j. Ich weiß es nicht

6. <u>Bitte wählen Sie die **beiden korrekten** Antworten zum Thema Impfung aus:</u>

    a. Lebendimpfstoffe sind unter Biologika kontraindiziert
    b. Können Infektionen verhindern
    c. Verschlimmern sehr oft Krankheitssymptome
    d. Ich weiß es nicht

7. <u>Bitte wählen Sie die **drei korrekten** Antworten aus der folgenden Liste aus:</u>

    a. Bettruhe für den größten Teil des Tages ist die beste Lösung, wenn Ihr Rücken schmerzt und steif ist
    b. Übungen zu Hause, Krankengymnastik, Massagen und Dehnübungen sind hilfreich, um Wirbelsäulensteifigkeit entgegenzuwirken
    c. Körperliche Aktivität und Training verschlimmern Müdigkeit (Fatigue)
    d. Müdigkeit (Fatigue) hat viele Ursachen: Entzündungen und Schübe, Medikamente, Lebensstil, Stimmung, Schlafstörungen

e. Es gibt eine individuelle Anfälligkeit für NSAIDs, und es sollte die niedrigste wirksame Dosis angestrebt werden
f. Ich weiß es nicht

Korrekte Antworten:

1	b,c,f
2	a,b,f
3	a,b,c,e,f
4	b,d,e,f
5	c,d,e,f,g
6	a,b
7	b,d,e
Summe der korrekten Antworten	__ / 25
